# A Scalable Architecture for the Dynamic Deployment of Multimodal Learning Analytics Applications in Smart Classrooms

**DOI:** 10.3390/s20102923

**Published:** 2020-05-21

**Authors:** Alberto Huertas Celdrán, José A. Ruipérez-Valiente, Félix J. García Clemente, María Jesús Rodríguez-Triana, Shashi Kant Shankar, Gregorio Martínez Pérez

**Affiliations:** 1 Telecommunication Software & Systems Group, Waterford Institute of Technology, X91 P20H Waterford, Ireland; ahuertas@tssg.org; 2 Faculty of Computer Science, University of Murcia, 30100 Murcia, Spain; fgarcia@um.es (F.J.G.C.); gregorio@um.es (G.M.P.); 3 School of Digital Technologies, Tallinn University, 10120 Tallinn, Estonia; mjrt@tlu.ee (M.J.R.-T.); shashik@tlu.ee (S.K.S.)

**Keywords:** smart classrooms, educational technology, multimodal learning analytics, internet of things, multisensorial networks

## Abstract

The smart classrooms of the future will use different software, devices and wearables as an integral part of the learning process. These educational applications generate a large amount of data from different sources. The area of Multimodal Learning Analytics (MMLA) explores the affordances of processing these heterogeneous data to understand and improve both learning and the context where it occurs. However, a review of different MMLA studies highlighted that ad-hoc and rigid architectures cannot be scaled up to real contexts. In this work, we propose a novel MMLA architecture that builds on software-defined networks and network function virtualization principles. We exemplify how this architecture can solve some of the detected challenges to deploy, dismantle and reconfigure the MMLA applications in a scalable way. Additionally, through some experiments, we demonstrate the feasibility and performance of our architecture when different classroom devices are reconfigured with diverse learning tools. These findings and the proposed architecture can be useful for other researchers in the area of MMLA and educational technologies envisioning the future of smart classrooms. Future work should aim to deploy this architecture in real educational scenarios with MMLA applications.

## 1. Introduction

Technology has been transforming education for the last decade. One of the main changes is the introduction of digital tools that support the learning and teaching practices [1]. Both software (e.g., smart tutoring systems, learning management systems, educational games, simulations, or virtual/augmented reality environments) and hardware (e.g., smart whiteboards, smartphones, remote labs, robots, wearable devices, cameras and other sensors) are present in the classroom and in our daily life [2,3]. The dynamism of classrooms requires the orchestration of this complex technical ecosystem, currently performed manually by instructors. Consequentially, novel technologies and mechanisms should be considered during the deployment of flexible and dynamic smart classrooms.

These rich ecosystems collect large amounts of data about the learning process and context, opening the door to better understand and improve education. However, handling such volume of raw data also represents a complicated challenge [4]. Aware of the promises and challenges, the area of Learning Analytics (LA) focuses on the “measurement, collection, analysis and reporting of data about learners and their contexts, for purposes of understanding and optimizing learning and the environments in which it occurs” (SoLAR definition of Learning Analytics https://www.solaresearch.org/about/what-is-learning-analytics). Within LA, over the last years, there has been a growing context on Multimodal Learning Analytics (MMLA), which is a sub-field that makes special emphasis on the usage of multimodal data sources [5]. There has been multiple and diverse MMLA applications, such as to teach how to dance salsa [6] or to assess oral presentations [7]. While transforming raw data into meaningful indicators is already daring [4], in this manuscript, we are mostly concerned with the issue of orchestrating the different data sources and applications. A recent literature review on MMLA architectures reveals that, due to the complexity of orchestrating the different elements of the technical ecosystem, most of the proposals offer ad-hoc solutions [8]. Apart from limiting the chances of reusability in different educational contexts, the effort to develop, deploy, maintain and enable interoperability among all those ad-hoc solutions does not scale up when the number of solutions increases [9]. Therefore, the current ad-hoc setup represents an important challenge to systematically apply MMLA in smart classrooms [10].

Thus, a real futuristic scenario with smart classrooms, where consecutive lessons take place (with 15–30 min breaks), would require a seamless and scalable reconfiguration of the sensors, devices and virtual learning environments within the classroom not only to deliver the lesson but also to profit from highly different MMLA solutions [10]. To address these challenges, we propose to evolve from traditional management, predefined by the instructor in a manual fashion, towards an automated approach able to reconfigure the classroom devices without human intervention and in a flexible and on-demand way. The number of sensors and actuators making up smart classrooms, as well as the possibility of managing them in a dynamic way make the scalability of the proposed approach a critical aspect to take into account. This can be possible by deploying a Mobile Edge Computing (MEC) architecture that combines Network Function Virtualization (NFV) technique [11] and Software-Defined Networking (SDN) paradigm [12]. NFV will allow for separating the software logic from the hardware of the classroom devices. It improves the flexibility and dynamism of device management processes by enabling the deployment, dismantling and reconfiguration of the technical ecosystem according to the current classroom needs. The SDN paradigm will help smart classrooms with automatic and dynamic management of network communications, enabling the Quality-of-Service (QoS) and interoperability of smart classroom devices and applications at the edge.

The objective of this paper is to present an MEC-enabled architecture that integrates SDN/NFV to deploy, configure and control the lifecycle of MMLA applications and devices making up a smart classroom as well as its network communications at any time and on-demand. More specifically, the objectives of this paper are as follows:

1. Use the MMLA literature to present a simulated but realistic scenario that can surface the limitations of the current technical approaches involved in the orchestration of complex technical ecosystems in educational practices.
2. Propose an MMLA architecture implementing SDN/NFV principles and exemplify how this architecture can solve some of the detected challenges to deploy, dismantle and reconfigure the MMLA applications in a scalable way.
3. Perform several experiments to demonstrate the feasibility and performance of the proposed architecture in terms of time required to deploy and reconfigure these applications.

The remainder of this paper is structured according to the next schema. Section 2 reviews and analyzes the state of the art of smart learning and classrooms, MMLA, remote smart classrooms, as well as the usage of SDN and NFV in different scenarios. Section 3 shows a case study explaining three different scenarios and their concerns. Section 4 describes the proposed architecture and how it can address the concerns of the aforementioned scenarios. Section 5 presents some experimental results that demonstrate the usefulness and performance of our solution. Section 6 discusses the main benefits of our solution compared to the existing ones. Finally, conclusions and future work are drawn in Section 7.

## 2. Related Work

### 2.1. Smart Learning Environments and Classrooms

In the last few decades, multiple terms have been coined with the “smart” label, often referring to devices (such as phones or watches) or spaces (e.g., classrooms, schools, campus, or cities) that through the utilization of the appropriate technologies and Internet of Things (IoT) services collect data from the users and the context to better adapt to the needs of the stakeholders involved. Aligned with this general idea, Smart Learning Environments (SLEs) are technology-enhanced learning environments able to offer instant and adaptive support to learners based on the analyses of their individual needs and based on the contexts in which they are situated [13]. Thus, when we think of a *smart classroom*, we should not reduce it to the mere idea of a traditional classroom heavily equipped with virtual learning environments and mobile, wearable or IoT devices.

While many aspects should be taken into consideration in a smart classroom such as the architectural design and its ergonomy, or the pedagogical methodology [14], in this paper, we focus on the infrastructure required to enable the “smart” features, i.e.,: (1) to seamlessly reconfigure such a complex technological infrastructure for guaranteeing the dynamicity and QoS of smart classrooms; and (2) to collect data from users and context to feed data for the intelligent adaptation to the learning needs at enactment time.

### 2.2. Architectures for Smart Learning Environments and Classrooms

As a recent literature review on smart campus technologies shows [15], paradigms and technologies such as the IoT, virtualization, wireless network, or mobile terminals are essential parts to be considered. There have been several attempts to orchestrate this intricate technical ecosystem. At the beginning, many of them were ad-hoc architectures suitable for specific technologies (e.g., interactive boards [16]), or focused on concrete problems (e.g., communication issues [17,18]) or features (e.g., remote software control [19]). Lately, authors have started broadening the scope and flexibility of their proposals. For example, GLUEPS-AR [20,21] combines the lessons learnt from distributed learning environments and the ideas coming from the MMLA domain. In [21], Serrano et al. designed an architecture which gathers student actions and their contextual data during across-spaces learning tasks to feed the adaption features. Another example is the architecture proposed by Huang et al. [22], which not only conducts the collection, integration and analyses of contextual data, but also enables the remote control of IoT devices and enhances the usability of the smart classroom with additional services such as voice recognition and user control interfaces. Previous colleagues also introduced LEARNSense framework [23], which aims to provide learning analytics using wearable devices. However, they did not deal with scalability and deployment issues either.

These architectures often focus on supporting data processing activities of the Data Value Chain (DVC) [24] (namely, collection and annotation, preparation, organization, integration, analysis, visualization, and decision-making). Each of these data processing activities poses a number of challenges linked to the problems associated with the data collection and analysis of multimodal data sources [8], which are common in smart classrooms. However, none of these proposals details how to (re)configure the smart classroom technical ecosystem to seamlessly switch from one LA application to another. Thus, in this paper, we try not only to enable the DVC in a smart classroom but also to reconfigure the technical ecosystem to cope with the requirements of different lessons happening in a row.

### 2.3. Remote Classrooms and Labs

Related to the technical orchestration challenges of smart classrooms, the virtual and remote lab field has a long trajectory coordinating IoT services and devices. Remote smart classrooms consider virtualization techniques and virtual machines (VM) to optimize the management of their software and hardware resources flexibly. Some remote laboratories consider virtual labs as an essential tool to improve the learning experience by supporting experimentation about unobserved phenomena [25]. In [26], the WebLab-Deusto project [27] used VMs to provide their students with remote smart laboratories that do not consider WebLab-specific code. Students had access to VMs for a given time and, once finished, a snapshot was made before restoring and preparing the VMs for new students. In [28], the authors proposed a solution that considered virtualization techniques to adapt the resources of remote laboratories at anytime and on-demand. Several experiments demonstrated how the usage of computing resources was optimized to guarantee the smart labs quality of service. In [29], the authors presented a mechanism to automatically generate, deploy and publish digitized labs in a framework of Massively Scalable Online Laboratories (MSOL). The authors demonstrated the suitability of the proposed mechanism by developing a communication protocol managing the lab equipment remotely, together with a web platform enabling the management of files and publishing digitized labs as web applications. Finally, the Smart Device Specification [30,31] provided remote labs with interesting capabilities. This specification focused on removing dependencies between clients and servers while enabling the description of remote lab experiments, and the selection of particular remote lab configurations [32]. However, configurations were not flexible enough because these must be established in advance by the lab administrator.

### 2.4. SDN and NFV Applied to Different Scenarios

The combination of SDN/NFV enables flexible, dynamic and on-demand management of networking and infrastructure resources. Moreover, it facilitates disruptive and heterogeneous scenarios such as the next generation of mobile networks (5G) [33], healthcare environments [34], or IoT [35].

Regarding 5G mobile networks, the authors of [36] analyzed the impact of SDN/NFV in the new vision of current and future network architectures. The authors highlighted how the combination of SDN/NFV reduces costs while improving the network flexibility and scalability of the infrastructure. The authors of [37] proposed a 5G architecture using NFV to support the implementation of tactile internet. A utility optimization algorithm which enables human perception-based tactile internet was developed to optimize the utility of 5G NFV-based components in this new scenario. In [33], the authors proposed an architecture which integrates SDN/NFV to manage and orchestrate services in charge of monitoring and controlling the network plane of a 5G network infrastructure in real-time and on-demand. Another solution was presented in [38], where authors studied the network flows migration of 5G networks and pointed out the inverse relationship between network load balancing and reconfiguration costs. Several experiments demonstrated the previous trade-off and the usefulness of the proposed solution. Regarding healthcare scenarios, the authors of [34] proposed an SDN/NFV architecture providing flexible and cost-efficient deployment and control of healthcare applications and services. In addition, the authors of [39] proposed an SDN/NFV framework to control the life-cycle and behaviour of physical and virtual medical devices belonging to clinical environments. This work also presented the novel concept of virtual medical device, an NFV-aware system providing dynamism in clinical environments. In the IoT context, the authors of [35] introduced an SDN/NFV architecture providing IoT devices with ultra-low communication latency. Another work was proposed in [40], where authors designed an architecture to ensure key security and privacy aspects of cyber-physical systems and IoT environments. SDN and NFV were considered to allow IoT devices and environments to make security decisions and take dynamic reactions. It is important to mention that learning scenarios such the proposed in this work can be improved by considering the SDN/NFV capabilities presented in the previous works.

In conclusion, this section has reviewed some of the most relevant solutions of heterogeneous smart learning environments and remote classrooms, highlighting the importance of seamless reconfiguration of smart classroom devices. The lack of solutions able to deploy, dismantle and reconfigure the software of classroom devices has also been demonstrated to ensure the seamless reconfiguration of devices in real-time and on-demand. Finally, we have shown the potential of SDN and NFV in other scenarios to achieve flexible and dynamic management of computational, storage and networking resources.

## 3. Description of Simulated Case Study

The related work review concluded that one of the main challenges in the area of smart classrooms and MMLA context is an architectural one. In our attempt to understand in depth this research issue, this section presents a simulated case study inspired by authentic uses cases extracted from the literature. The main goal is to ascertain what the specific issues are that our proposed architecture must address (see in next Section 4), in order to support a seamless reconfiguration of a smart classroom where different learning activities would happen in a row. With the objective of building this case study, we reviewed literature on MMLA applications that have been implemented during the last few years. From these cases, we select three that were aligned with innovative learning trends and have different objectives, devices, analytics and sensors in order to demonstrate how the architecture self-organizes from one scenario to the following one. We also order these three cases by increasing complexity, the first one focuses on individual students, the second one focuses on groups of students collaborating, and the third one focuses on students collaborating in projects but also on what the instructor is doing. Next, we describe in depth each one of the scenarios.

### 3.1. Intelligent Tutoring System in the Classroom

One of the main trends in education over the last decade has been the development of interactive environments that can be slowly introduced as part of the classroom or homework activities. Two of the most relevant tools for this purpose are Intelligent Tutoring Systems and Educational Games [41]. Most of the literature meta-reviews that have measured the effectiveness of such tools in the classroom [41,42] have reported positive effects. However, these studies also agree on the struggle that instructors face to effectively integrate these tools in their teaching and curriculum. One of the reasons is not being able to know what students are doing in these virtual environments to orchestrate the classroom activities and to intervene if necessary. Hence, the need for the development of real-time dashboards that can provide this information to instructors [43].

The first scenario is grounded in this technological and pedagogical issue, and is strongly inspired in the previous work of Holstein et al. [44,45]. In this work, they have co-designed a dashboard and augmented wearable instruments to visualize real-time analytics and visualizations of what each student is doing in the intelligent tutoring system. Next, we detail the specific details:

- **Context**: In this scenario, students are practicing a specific topic through the use of an intelligent tutoring system. Each student is individually interacting with the environment with the computer. In order to provide just-in-time help, instructors need to know how students are advancing in this practice and what are their mistakes or misconceptions. A usual class would be around 20 to 40 students.
- **Application**: When students interact with the intelligent tutoring environment, they generate events and clickstream data that can be processed to make inferences about their learning process. Based on these data, the analytics engine generates a number of indicators of students’ current skill and behavioral states. For example, it can show if a student is confused, needs help, has been idle for a number of minutes or their areas of struggle, among other pieces of information. Additionally, each computer has a webcam capturing students’ face and expression, and the analytics engine applies an affect detection Machine Learning (ML) model to infer students’ affect status. Instructors receive all these info through a dashboard in real-time and can easily move within the classroom attending students’ needs.
- **Sensors and devices**:
  – *Individual students’ devices*: Students interact with the ITS by connecting to it as a web application. The ITS provides of a series of scaffolded exercises adapted to the current level of skill of each student. Students use the desktop PC available in the classroom.
  – *Individual students’ webcam*: Each student has a front camera in their computer that is capturing a video feed of their face expression continuously. This feed is used by the analytics engine to infer the emotional state in time windows.
  – *Instructor device*: The instructor consume the analytics via a dashboard by connecting from its device (tablet or laptop) to the visualizer provided by the architecture.

### 3.2. Tabletop Task Collaboration

UNESCO has noted that the future of education should be focused on promoting transverse skills, such as collaboration [46]. The trend has shifted from individual efforts to group work, making the development of collaboration skills mandatory with an increasing trend of implementing collaborative learning activities with high frequency [47]. Therefore, it is not a surprise that numerous researchers have started to analyze collaborative learning from different perspectives. However, one of the challenges has been to scale up the analysis of these collaboration studies when there are many groups to assess or to provide feedback in real-time. Hence, the area of MMLA has been studying ways to automatically provide empirical evidence that can help to support co-located collaboration through analytics [48]. In these studies, researchers capture multimodal data from the collaboration, some examples of data sources include video, audio, physiological signals using wearables or interaction data with computers or shared devices [49,50].

This second scenario is grounded in this context where we present an application that generates colocated collaboration analytics while students are interacting on a multi-touch tabletop doing a collaborative task, which is based on previous work from Maldonado et al. [51]. The details of this scenario are depicted next:

- **Context**: In this case scenario, we have students interacting with a shared device known as interactive multi-touch tabletop, which can easily support face-to-face collaboration with multiple students interacting at the same time. Students carry out an activity on collaborative concept making, which is a technique where learners represent their understanding about a topic in a graphical manner by linking concepts and preposition [52]. At the same time, students are also conversing with each other and discussing their decisions, and this voice stream is also captured through a microphone. The class is organized in groups of three students, and a usual class could have around 7 to 14 groups.
- **Application**: The objective is to design an application that can help teachers become more aware of the collaborative process, by making visible interactions that would otherwise be hard to quantify or notice. The application study collaboration by considering both the verbal interactions when students are talking to each other, as well as physical touches with the table-top [53]. More specifically, it can use metrics to identify learners that are not contributing enough to the activity or are dominating it (both physical and verbal interaction), groups that can work independently or those that do not understand the task. The instructor accesses all these information though a visualization dashboard in a hand-held device.
- **Sensors and devices**:
  – *Group multi table-top*: Table-top learning environments are big tactile screens that allow the collaboration of multiple users at the same time.
  – *Group overhead depth sensor*: A Kinect sensor is used to track the position of each user automatically detecting which student did each touch.
  – *Group microphone array*: It is located above the tabletop and captures the voice of all the group members, distinguishing the person which is speaking.
  – *Instructor device*: The instructor consume the analytics via a dashboard by connecting from its device (tablet or laptop) to the visualizer provided by the architecture.

### 3.3. Programming Project-Based Learning and Instructor Indoor Positioning

Project-based learning has become one of the main forms of instructions across contexts and the different phases of schooling as it resembles better real-world practices and leads to deeper learning [54]. This method of instruction is very common in programming courses, where students often have to develop a collaborative group programming project to pass the course (e.g., [55]). One of the challenges of these collaborative projects is to assess the role and effort of each member of the group in order to guarantee similar workload distribution, hence avoiding free riding [56]. These project-based learning courses often have entire sessions devoted to in-class work on the projects. During these sessions, the teacher moves from group to group solving doubts, which presents a new challenge regarding how to equitably distribute their time across groups [57]. In this context, we can collect diverse sources of data from the collaborative programming environments, audio from group conversations, instructors’ position and physiological signals from the students.

This third scenario combines inspiration from the following previous studies: the work of Spikol et al., and Blikstein to apply MMLA to analyze collaborative project-based learning and open-ended programming tasks [58,59], the ideas of Ahonen et al., to analyze biosignals during these programming tasks [60] and finally the proposal of Martínez-Maldonado et al. to estimate the amount of time spent by the instructions in each group [57]. Therefore, in this scenario, the application combines an analysis of the collaborative programming actions and conversation of each group, the physiological signals levels of each student and position of the instructor. More details about this scenario are depicted next:

- **Context**: Numerous programming courses have capstone projects where students need to implement an application that shows evidence of the different concepts acquired thorough the course. These courses usually have some sessions allocated for students to start developing these projects in groups while instructors move from one group to another solving doubts. Each group interacts with a shared programming environment (e.g., [61]) to develop the project collaboratively. The class is organized in groups of three students, and a usual class could have around 7 to 14 groups.
- **Application**: In this scenario, there are two main applications. The first one is to provide analytics regarding how the collaboration is working out and how the project is advancing. This can include information regarding areas of struggle based on the code written and code compilations [59], but also regarding the level of contribution to the project of each member, analysis of the conversation and engagement levels obtained through the analysis of the physiological signals to measure activation and engagement levels. The second one is an automatic control of how much time the instructor has spent helping each one of the groups through indoor positioning; this way, the instructor can balance the help that each group receives. The instructor can consult all this information through a dashboard in order to provide just-in-time and personalized support to each group.
- **Sensors and devices**:
  – *Individual students’ devices*: Students interact with the collaborative programmings environment by connecting to it through a web application.
  – *Individual Empatica E4 wristband*: Each student wears an E4 empatica wristband that captures the heart rate, a three-axis activity through an accelerometer, and the electrodermal activity of their skin.
  – *Group microphone array*: It is located above each one of the groups’ tables, distinguishing the person which is speaking.
  – *Group positioning sensor*: It is located in each one of the groups’ tables to detect the center position of each group.
  – *Instructor positioning badge*: It is carried by the instructor when moving around the class. It implements Pozyx (https://www.pozyx.io/) technology which is an ultra wide band solution that provides accurate positioning and motion information with sub-meter accuracy (10 cm).
  – *Instructor device*: The instructor consumes the analytics via a dashboard by connecting from its device (tablet or laptop) to the visualizer provided by the architecture.

### 3.4. Requirements of the Previous Scenarios

The case study with the three consecutive scenarios represents an example of how smart classrooms and MMLA solutions could look in the future. To reach our goal of supporting the seamless reconfiguration and data collection required to enable the smart adaptation, we have identified four main requirements emerging from our simulated case study:

**Requirement 1—Within-scenario flexibility for instructor-configured data collection, analytics, visualizations, and recommendations**: Aligned with the challenges reported in the literature [8], the MMLA solutions implemented in the aforementioned scenarios are ad-hoc solutions that enable the data gathering and analysis to later feed the visualizations and recommendations for instructors and students. The three use cases that we described have different learning environments, devices, data sources or analytics pipelines that have been configured to match the necessities of each use case. Therefore, to be able to scale up the number of MMLA solutions used in a single classroom and scenario, it is necessary to provide a scalable architecture compatible with the different MMLA applications [9,10] by abstracting these functionalities in scalable and interoperable modules that can be automatically re-configured for each MMLA application.

**Requirement 2—Between-scenario flexibility for automatic deployment of the MMLA solutions**: The kind of equipment, devices, setup and sensors necessary to perform these applications makes smart classrooms expensive to have. Therefore, we would expect that, in the future, these classrooms are fully booked, perhaps having a short time of 15–30 min in-between sessions. In our case study, we presented three consecutive use cases to illustrate this issue, but this might be a conservative estimate. The current setup makes it very challenging to seamlessly and automatically re-configure the technical ecosystem and to also enable the data collection and analysis in short periods of time. In our case study without a proper architecture, each teacher would be in charge to deal with the technological complexity of the MMLA application in each class, which in reality is not a feasible approach. This raises the necessity to have a seamless transitions between the scenarios of our simulated case study.

**Requirement 3—Seamless privacy and authentication configurations**: The privacy of users, and of students in this case scenario, has been one of the topics on the spotlight during the last years [62]. The regulations have agreed that we need to provide control to the users so that they can specify how their data can be used. Therefore, even though these MMLA solutions seek to help students in their learning process, students and instructors should still have the right to opt-in or -out so that their data are not collected and/or used. In the case scenario, each application would need to manage this privacy and authentication issues separately, which is sub-optimal. Therefore, we need to provide a centralized system where students can configure their privacy and authentication options to apply across all the smart classroom applications, and we also need to easily identify students across applications and devices so that we can properly process their data.

**Requirement 4—Easy communication with external data sources**: Thanks to the institutional data and the ICT adoption in our daily routines, there can be numerous data sources (both formal and informal) that can hold valuable information to understand students’ context and knowledge. Some examples might include the classical LMSs in formal learning institutions, other online courses, academic records or background information. In the case study, each application would have developed their own interface to interact with these external data sources. Thus, instead of implementing ad-hoc solutions to benefit from those external data sources, there is a need for generating services and APIs that can be used across applications.

## 4. Architecture

**Figure 1:**
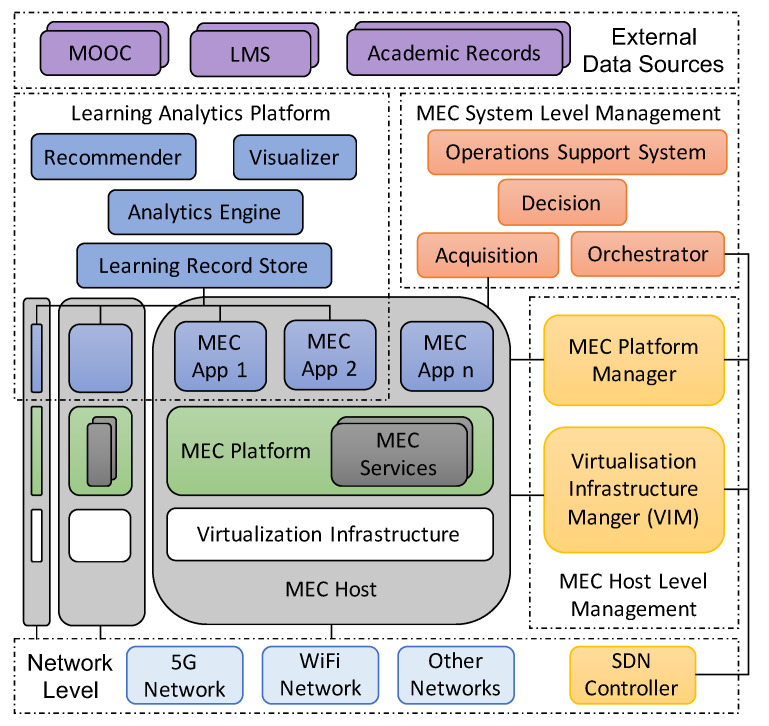
Architecture oriented to the Mobile Edge Computing (MEC) paradigm.

This section describes our MEC/SDN-oriented architecture that satisfies the aforementioned requirements, and how it integrates different components to reconfigure and manage the learning applications running on top of classroom devices automatically, on-demand and in real-time. Figure 1 shows the levels, components and communications of the proposed architecture. The main elements, following a top-down approach, are the next ones:

- *External Data Sources*. This level contains different external databases and tools such as data from the Academic Records, Learning Management System (LMS) or Massive Open Online Courses (MOOC) that can feed our architecture with relevant students’ data.
- *Learning Analytics Platform*. It hosts the components focused on analysing data provided by external sources and generated during the realization of learning activities.
- *MEC System Level Management*. This level is focused on (1) processing requests from instructors to reconfigure heterogeneous classroom devices in real-time and on-demand, (2) making decision and orchestrating them to configure learning applications running on top of classroom devices, and (3) sensing classroom devices to detect misconfigurations or problems.
- *MEC Host*. Heterogeneous classroom devices, also known as MEC Hosts, such as electronic blackboards, tablets, personal computers, servers, or Raspberry Pi that need to be reconfigured according to the current learning course or subject.
- *MEC Host Level Management*. Level hosting the different managers able to control the life-cycle of the Virtualization infrastructure, MEC Platform, and MEC Apps running on the MEC Hosts.
- *Network Level*. This level contains the network infrastructure enabling the communication of MEC Hosts and the rest of the levels making up the architecture.

In the following subsections, we explain in detail the components and main levels of our platform.

### 4.1. Learning Analytics Platform

The *Learning Analytics Platform* has the different modules and components that are necessary to implement learning analytics applications that have as a final objective to improve the learning experience and outcomes of students. With that goal in mind, the platform hosts different components able to acquire, process, analyze, recommend and visualize relevant data generated during the interaction of students with learning applications. Among the most relevant components, we highlight the *Learning Record Store (LRS)*, which acquires and stores students’ interaction registers generated by learning applications. Those registers are sent to the *Analytics Engine* component to analyze them by using ML and statistical techniques. According to the registers, the outputs of the *Analytics Engine* and some trained models, the *Recommender* component provides students and instructors with suggestions to improve the learning experience. Finally, the *Visualizer* component exposes a graphical interface that allows students and instructors to interact with registers, data and outputs of the learning platform.

### 4.2. MEC System Level Management

The *MEC System Level Management* deals with the management of the classroom devices and the behaviour of the learning applications running on top. In this context, the *Operation Support System* (OSS) is focused on the the logic of the architecture. This element provides instructors with an interface to define the rules that enable the reconfiguration of the learning applications and software running on top of the heterogeneous devices belonging to a classroom. These rules will be provided to the *Decision* component to identify particular actions to be taken. Once a decision is made, the *Orchestrator* receives the notification and interacts with the managers and controllers of the lower levels to configure the network, the classroom devices and their learning applications. Finally, the *Acquisition* component senses data generated by the classroom devices and their applications and services (not only learning applications) to detect misconfigurations or problems. When one problem is detected, the *Decision* and *Orchestrator* modules come into play to decide, schedule, and spread the required actions.

### 4.3. MEC Host Level

The *MEC Host Level* is composed of two planes, the control and data planes.

The control plane is called *MEC Host Level Management* and it is in charge of deploying, controlling and dismantling learning applications, instantiated as *MEC Apps* that run on top of heterogeneous classroom devices (*MEC Hosts*). The MEC Host level management contains two managers: the *MEC Platform Manager* and the *Virtualization Infrastructure manager* (VIM). The MEC Platform Manager controls the whole life-cycle of MEC Apps, and the VIM manages the computation, storage and networking virtual and physical resources of the Virtualization Infrastructure.

In the data plane, we find the MEC Hosts, which are classroom devices providing computational, storage, and networking resources to execute learning applications. Each MEC Host contains a *Virtualization Infrastructure*, a *MEC Platform* and one or more *MEC Apps*. MEC Apps can be deployed as learning applications, components of the Learning Analytics Platform (commented on in Section 4.1) and other applications like, for example, those oriented to improve the learning courses security and privacy). MEC Apps can be instantiated in Virtual Machines (VM) or containers running on top of the virtual infrastructure. The virtualization infrastructure consumes the hardware of heterogeneous learning devices such as computers, digital blackboards, or cameras and provides computational, storage and networking virtual resources. Finally, the MEC platform provides essential and generic MEC Services needed to run MEC Apps. These services can be specific for particular applications or generic enough to be shared among several MEC Apss. Examples of MEC Services can range from communication protocols to access control mechanisms or cryptographic material.

### 4.4. Network Level

The *Network Level* contains two types of elements: heterogeneous *Networks* and the *Network manager*. The networks represent the hardware and software networking resources needed to connect MEC Hosts and their MEC Apps. The Network Manager allocates the SDN Controller, which has the global view of the network status as well as the logic of the network to control the data plane where heterogeneous networks are located.

### 4.5. Solutions Provided by our Architecture to the Previous Requirements

**Figure 2:**
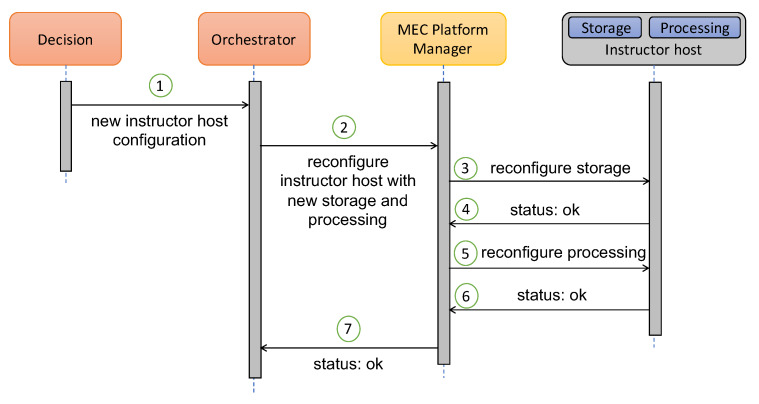
Architecture reconfiguring two MEC Apps running on top of an MEC Host.

**Solution to Requirement 1—Within-scenario flexibility for instructor-configured data collection, analytics, visualizations and recommendations**: Easy and flexible reconfigurations of the instructors’ and learners’ applications, such as the one needed in the first scenario, are enabled by our solution. Figure 2 shows the interaction between the components of our architecture to reconfigure the storage and processing capabilities of the instructor host. For clarity’s sake, we show how the architecture reconfigures two MEC Apps running on top of an MEC Host. However, this functionality could be extended to several MEC Hosts and applications. The 1st step of Figure 2 shows when the decision of reconfiguring the instructor host is made by the Decision component. After that, the Orchestrator provides the MEC Platform Manager with the MEC Host and the reconfiguration details of the new storage and processing capabilities. Once received, the MEC Platform Manager interacts with the instructor host to access the storage and processing MEC Apps and reconfigure them (steps from 3 to 6). When the reconfigurations have finished, the action is confirmed to the Orchestrator (step 7).

**Figure 3:**
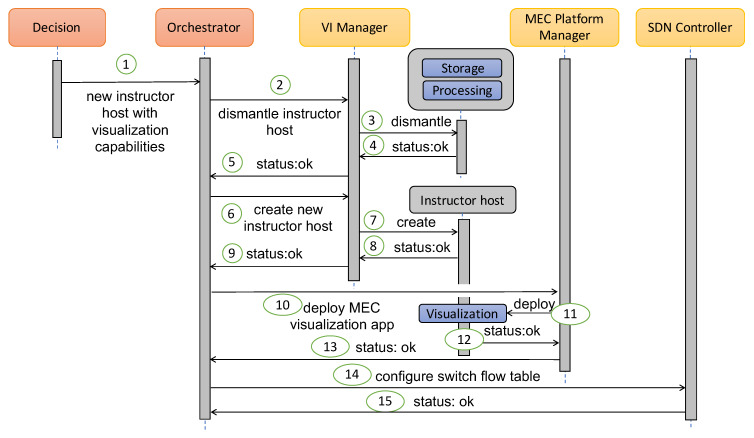
Architecture dismantling an old MEC Host, and deploying a new MEC Host and MEC App.

**Solution to Requirement 2—Between-scenario flexibility for automatic deployment of the MMLA solutions**: Aligned with the capabilities shown in the previous issue and focused on addressing this one, the proposed architecture deploys, configures and dismantles MEC Hosts and their applications in real-time and on-demand. Following the previous example, Figure 3 shows how the components of our architecture dismantle the instructor host when a given application is finished, and deploy new ones with different capabilities for the next class. In the 1st step of Figure 3, the Decision component interacts with the Orchestrator to notify the necessity of changing the instructor host. After that, the Orchestrator provides the VI Manager with the required info to dismantle the MEC Host (step 2). Once the notification is received, the VI Manager dismantles the host and confirms the Orchestrator the action (steps 3–5). When the old instructor host has been dismantled, the next step is to deploy a new MEC instructor host with more hardware resources (processor and graphics). This process is shown from 6 to 9 in Figure 3. At this stage, our architecture has already deployed a new MEC instructor host with enough hardware resources to meet the requirements of the next learning analytics application and the next step is to deploy a new MEC App with visualization tools and capabilities. For that, the MEC Platform Manager is the component in charge of deploying, configuring and confirming the new MEC App (steps from 10 to 13). Finally, the Orchestrator communicates with the SDN Controller to include a new rule in the switch flow table, and route the network packets sent and received by the new instructor host and its applications (step 14).

**Solution to Requirement 3—Seamless privacy and authentication configurations**: Our architecture is able to deploy MEC Apps, providing students with authentication and authorization capabilities, in real-time and on-demand. On the one hand, depending on the learning course security requirements, the architecture will deploy and configure an MEC App providing several authentication mechanisms with different levels of security. On the other hand, the architecture will deploy another MEC App allowing students to define their privacy preferences by defining user-friendly policies. In this context, students will determine what pieces of sensitive data can be shared, who or what learning tools can process the sensitive data, how long data can be processed or stored, or what can be done with the data, among others. Once defined the policies, they will be sent to the components of the Learning Analytics Platform to ensure that they are considered during the data management and storage processes.

**Solution to Requirement 4—Easy communication with external data sources**: As can be seen on top of Figure 1, the design of our architecture considers external data sources such as MOOC, LMS, or academic datasets feeding the Learning Analytics Platform with additional data that will be critical for the data analysis processes performed by its components.

## 5. Experimentation Results

A key aspect of our proposal is how the architecture deploys and configures the learning ecosystem automatically for each scenario, which addresses the aforementioned Requirements 1 and 2. We consider these two requirements as the key ones that are necessary to bring scalability and interoperability to smart classrooms and MMLA applications, and thus we focus our experimentation in this section on those two aspects. The deployment process is dealt by the Orchestrator that must consider the features of each classroom device and its performance with different MEC Apps. In this section, we show experimental results regarding computational performance and efficiency of typical classroom devices with practical learning tools.

With a model of deploying MEC Apps based on containers, we investigate experiments about three types of learning tools: high-intensive computing, medium-intensive computing and high-intensive data consuming. The high-intensive computing MEC App is a face-recognition that detects all the faces and face encoding in each frame of a video source. This application is a Python program based on *dlib* library using a Histogram of Oriented Gradients (HOG) face detector. The medium-intensive computing MEC App is a feature extractor for Automatic Speaker Recognition (ASR). This application is also a Python program based on the Mel-Frequency Cepstral Coefficients (MFCC) that analyzes an audio source periodically each second. In addition, the high-intensive data consuming MEC App is a computational physics simulation that plots a 3D surface. This application is a Python program based on Matplotlib library for creating animated visualizations.

### 5.1. Testing Environment

We deployed a testing environment composed of three MEC Hosts with different hardware resources, which are representative of a real smart classroom; these are a server, a desktop PC and a laptop. These devices can be used in the different scenarios presented in Section 3. The server was an Intel machine with dodeca-core (12 cores) 3.50 GHz CPU and 32 GB DDR4 RAM, the PC was an Intel machine with octa-core (8 cores) 3.40 GHz CPU and 16 GB DDR4 RAM, and the laptop was an Intel Celeron machine with dual-core 1.10 GHz CPU and 4 GB DDR4 RAM. In particular, laptops have similar computational capabilities to tablets and mini-PCs, so our experimental results with laptops are comparable to tablets and mini-PCs.

For each device, we set up a realistic evaluation environment with the typical services and graphical interface used to reduce the overhead. The operating system of all hosts was Ubuntu 64-bit 18.0.4, and the containers were deployed by the latest version (19.03.6) of Docker Engine. No more additional software components were needed to deploy the learning tools on our testing environment. Each learning tool was allocated within a unique Docker container providing a single learning task.

Our testbeds evaluated the performance and efficiency of our solution by increasing the number of containers on each type of MEC Host. This allows for observing the performance variance across different scenarios according to their capabilities. We expect that changing between scenarios would have an impact in the performance, e.g., the learning device installed in a classroom work table would require much more learning tools in a Tabletop Task Collaboration scenario than a Programming Project-based Learning scenario. Another possibility is that there could be changes in the number of students taking each class, hence affecting the computation requirements. Therefore, the performance for each configuration must be well-known by the Orchestrator to properly reconfigure the learning devices in each class.

### 5.2. Docker Container with High-Intensive Computing Application

There are several learning scenarios that can require a face detection tool to identify students or infer affect states. As shown in Section 3 for an Intelligent Tutoring System, an MEC Host with a camera capturing a video feed of student face expression can be used to infer the affect (e.g., surprise, neutral, confusion and angry) and identify when a student needs help. We used *dlib* library to implement a HOG face detection MEC App and created a Docker container that provides this app in our testing environment. The HOG is one of the most reliable and applied algorithms for person identification, but also an intensive computational task. Therefore, it is essential to properly manage the available computing resources in the learning device that can be dedicated to the execution of this learning tool.

**Figure 4:**
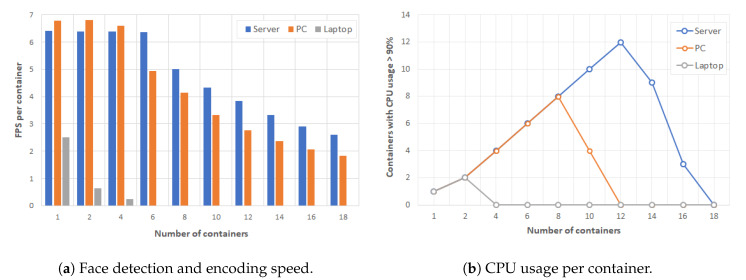
Performance results for Face Recognition application in Docker containers.

In order to evaluate the performance and efficiency of the Face Recognition application in Docker containers, our testbed used a H.264 video source with 640 × 360 image size and applied the HOG algorithm in each video frame. We used the analyzed frames per second (FPS) as performance evaluation index to assess how fast the HOG algorithm is. If a configuration has higher FPS value, it has higher video quality and can produce smoother video. Figure 4 shows the experimental results obtained when increasing the number of containers for each type of learning device. The left graph depicts the maximum analyzed FPS for each configuration and the right graph shows how many CPU cores are overloaded.

As it can be seen in Figure 4, the maximum speed achieved was above 6 FPS for configurations with up to six containers in server and up to four containers in PC, whereas the throughput in the laptop was much lower with less than 3 FPS. In addition, the server was absolutely overloaded with 12 containers, the PC with eight containers and the laptop with two containers. Therefore, we observe that each container consumed approximately one CPU core. These experimental results imply that a face detection tool can be provided in different configurations e.g., a PC with eight cameras could serve for a work table shared by eight students or a laptop for one single student. Note that the server achieved the highest computation performance, and this performance could further improve if it included a graphics card to implement the HOG algorithm.

### 5.3. Docker Container with Medium Computing Application

Identifying students via their voice in a microphone can be useful for several learning scenarios, as shown in our use case related to project-based learning (see Section 3). An MEC Host with a microphone capturing the meeting audio can identify students, perform speech-to-text transcription, calculate speaker metrics (e.g., speaking time or counters) and infer the emotional state (e.g., angry, boring or excited).

We implemented an MEC App based on MFCC to recognize persons and created a Docker container with this tool to carry out our experiments. The MFCC are widely used in automatic speech and speaker recognition and allow transforming the audio source into a sequence of feature vectors that characterize voice signals. Our MEC App extracted feature vectors in one second window in order to apply a real-time student recognition. The process to calculate MFCCs consisted in framing the signal in short windows to later apply specific mathematical operations that convey a medium computing task.

**Figure 5:**
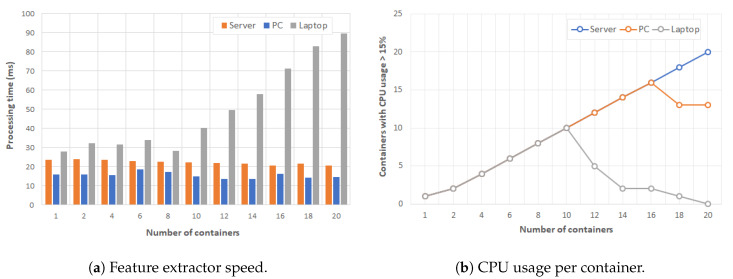
Performance results for Automatic Speaker Recognition application in Docker containers.

In order to evaluate the performance and efficiency of the ASR application in Docker containers, our testbed used an audio signal stored and processed each second using MFCC with a length of the analysis window of 25 ms and a step between successive windows of 10 ms. In this case, we used the processing time as the performance evaluation index because this indicator shows how fast the MFCC algorithm is. If a configuration has slower processing time, it can process more audio sources and serve more users. Figure 5 shows the experimental results obtained when increasing the number of containers for each type of MEC Host. The left graph depicts the processing time to analyze the audio signal each second and the right graph shows how many CPU cores are used in the processing.

The audio feature extractor is a relatively low computationally expensive task that is well-supported in server, PC and even laptop. As shown in Figure 5, the processing time was always below 100 ms for our three learning devices and below 30 ms for server and PC. However, the CPU overload was relevant for PC when the number of containers doubles its number of cores. In addition, the laptop was stuck when the number of containers was greater than 10, whereas the server was not overloaded with up to 20 containers. These experimental results show that an ASR tool can be easily provided in our use cases, e.g., a laptop/tablet with microphone could serve a 6-student work group or a server with 20 students simultaneously.

### 5.4. Docker Container with a High-Data Consuming Application

The interactive simulation-based learning can be useful in multiple scenarios, for example using an ITS in the classroom, as shown in Section 3. When students interact with the simulation, they generate events and clickstream data that can be stored and processed to calculate usage metrics (e.g., idle times or event counters) and even infer about their learning experience (e.g., difficulty or simplicity).

There are several types of interactive simulations which could be used in a classroom. Physics simulations are widely used to improve the learning process in science and engineering education. We implemented a Matplotlib MEC App to build an animated physics simulation that shows a wave motion. In particular, the physics simulation used a 1.5 GB array to plot a 3D surface animated. The size of plotting array implied that the simulation carried out a high data consuming task for learning devices or MEC Hosts.

**Figure 6:**
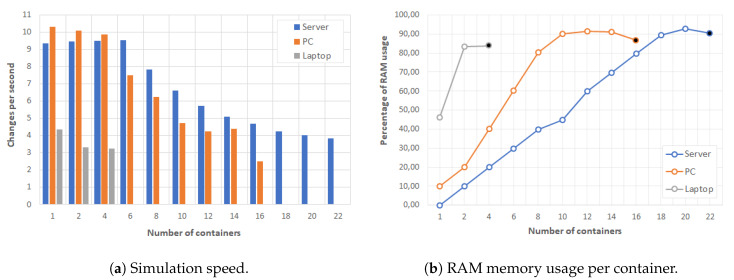
Performance results for Computational Physics simulation in Docker containers.

In order to experiment our physics simulation in Docker containers, our testbed updated the plotting constantly in order to evaluate the performance in each learning device. We used the changes per second (CPS) of the simulation as the performance evaluation index because this indicator shows how fast the simulation is running. If a configuration has higher CPS value, it has higher simulation quality and can produce more fluent simulations. Figure 6 shows the experimental results obtained when increasing the number of containers for each type of learning device. The left graph depicts the maximum CPS for each configuration, and the right graph shows the percentage of RAM memory used.

Given the results shown in Figure 6 and that our physics simulation required 4 CPS at least to show a fluent animation, a laptop served only one container with our simulation. However, the server and PC could achieve 20 and 14 containers, respectively. Moreover, the memory was full and additional containers were rejected when the server launched more than 20 containers, the PC 14 containers, and the laptop 2 containers. These experimental results show that a high-data consuming simulation can be used in different configurations, e.g., a laptop/tablet could be used for a single student and a server to up to 20 students in the classroom.

## 6. Discussion

Among the different aspects to be taken into consideration in a smart classroom [14], the proposed architecture focuses on orchestrating the complex technical ecosystem and enabling its “smart” features. The architecture has been designed bearing the following main requirements in mind: within-scenario and between-scenario flexibility, seamless privacy and authentication configurations, and easy communication with external data sources.

The experimental results regarding the performance and scalability of our architecture show how heterogeneous classroom devices can be managed in an automatic and efficient way to host different amounts and types of learning tools and applications. Concretely, we demonstrated the scalability of our architecture when an increasing number of Dockers, with diverse computational requirements, is deployed over three widely used hardware configurations such as laptops, personal computers and servers. However, no direct comparison of the obtained results with those reported in the literature was possible since they highly depend on the hardware and software configuration. Furthermore, most MMLA studies evaluate their results based on educational outcomes but not on technical performance.

The automatic and flexible management of the proposed architecture has been motivated through the case study presented in this paper, which illustrates the limitations of current solutions and how our proposal offers a seamless switch between three different learning scenarios happening in the same smart classroom. While existing architectures for smart classrooms often involve ad-hoc digital devices and tools that can be used in specific ways [15,22], in our proposal, the different modules of the ecosystem can be orchestrated for multiple purposes in scalable and interoperable ways. Moreover, the human intervention required to adapt and reconfigure the transition between heterogeneous learning lessons is significantly reduced and can be automatized.

The presented architecture could be of great value also for the remote lab community. While virtualization techniques had been already explored [27,28,29], this architecture could increase the flexibility of remote labs, by supporting the configuration and deployment of remote experiments [32]. Moreover, it supports the collection of multimodal data (coming both from hardware and software) necessary to support the smart adaptation to the learning process.

Regarding the instant and adaptive support expected from smart classrooms [13], our proposal could become the base upon which other architectures could build, uncoupling the multimodal challenges of the DVC [4,63]. More concretely, our contribution helps to address the lower level technical requirements of the DVC, and more conceptual architectures (e.g., [21,22,63]) could build on top of it. Thus, our proposal contributes to diminishing the need for ad-hoc MMLA solutions often due to the technical constrains to the ecosystem [8]. As a consequence, relying on a lower level architecture will open the door to multiple analysis and adaptability schemes in smart classrooms, addressing the reusability and interoperability problems among MMLA solutions [9,10].

The integration of SDN/NFV in our architecture allows instructors to reduce their workload avoiding the manual configuration of classroom devices according to the topic and purpose of each subject. It also reduces the complexity of the smart classrooms management as well as optimizes the usage of classrooms devices. In a nutshell, smart classrooms equipped with our architecture will be able to reconfigure and optimize the learning applications of their devices ant their communications according to the current subject topic and number of students. It will be done in real-time and on-demand. In contrast, as it has been demonstrated in Section 2, existing solutions using virtualization techniques [25,26] are not able to reconfigure the whole remote lab in a flexible way. They just consider predefined VMs implementing particular learning applications that are instantiated and dismantled. It means that they miss critical aspects such as the flexible management of communications, essential to guarantee QoS issues when the number of students increases, and the optimization of hardware resources of learning devices such as CPU, memory and storage.

It is important to note that one of the main limitations of the proposed architecture is the complexity of its deployment. The usage of resource-constrained devices such as digital boards or cameras makes very complex their management through current virtualization techniques. Fortunately, this issue is limited when other devices such as tablets and personal computers are considered in smart classrooms. Additionally, the architecture is still to be tested in a real scenario, which is part of the future work. Moreover, we argue that the architecture represents an improvement with respect to other studies. However, we cannot present a direct comparison in terms of efficiency because most MMLA studies do not report on the performance of the architectures from the technical point of view. Finally, we still have not tackled the challenge of how instructors will be able to interact with this architecture through a user-friendly authoring tool.

## 7. Conclusions and Future Directions

Smart classrooms require a dynamic and flexible orchestration of their complex ecosystem, currently performed manually by instructors that use ad-hoc learning applications. With that goal in mind, this paper the following three key research problems: (1) the limitations of current learning solutions in terms of flexible and scalable management of devices belonging to simulated and realistic learning scenarios; (2) the suitability of technologies and their integration in an architecture able to provide the level of flexibility and dynamicity required by current learning environments; and (3) the scalability and performance of the architectures. With challenges in mind, this paper proposes an MEC-enabled architecture that considers SDN/NFV to reconfigure the software and hardware resources of classroom devices in real-time and on-demand. A case study inspired by authentic learning analytics applications extracted from the literature has been proposed to highlight the limitation of the existing solution and demonstrate the added value of our architecture. The experimental results demonstrate acceptable computational performance and efficiency when typical classroom devices such as servers, personal computers or laptops implementing practical learning tools are deployed and reconfigured. Specifically, we investigated experiments with different MEC Apps such as face detector, ASR and physics simulation, each one with different computational requirements. The results point out the potential of our architecture to manage heterogeneous classroom devices in an automatic and efficient way.

As future work, we plan to implement and deploy the proposed architecture in a realistic smart classroom scenario to demonstrate its usefulness with real students. In this sense, we will integrate our architecture in existing platforms able to deploy, dismantle and control the life-cycle of VMs and containers such as OpenStack, as well as control the network infrastructure and the communications of the smart classroom by using OpenDaylight as SDN Controller.
